# Pancreatic β-Cell O-GlcNAc Transferase Overexpression Increases Susceptibility to Metabolic Stressors in Female Mice

**DOI:** 10.3390/cells10102801

**Published:** 2021-10-19

**Authors:** Ramkumar Mohan, Seokwon Jo, Elina Da Sol Chung, Eunice Oribamise, Amber Lockridge, Juan E. Abrahante-Lloréns, Hai-Bin Ruan, Xiao-Yong Yang, Emilyn U. Alejandro

**Affiliations:** 1 Department of Integrative Biology and Physiology, University of Minnesota, Minneapolis, MN 55455, USA; rammohan@med.umich.edu (R.M.); joxxx057@umn.edu (S.J.); elina.dasol.chung@gmail.com (E.D.S.C.); oriba004@umn.edu (E.O.); lockr008@umn.edu (A.L.); hruan@umn.edu (H.-B.R.); 2 Supercomputing Institute, University of Minnesota, Minneapolis, MN 55455, USA; abrah023@umn.edu; 3 Program in Integrative Cell Signaling and Neurobiology of Metabolism, Yale University, New Haven, CT 06520, USA; xiaoyong.yang@yale.edu

**Keywords:** O-GlcNAc transferase, islet, streptozocin

## Abstract

The nutrient-sensor O-GlcNAc transferase (Ogt), the sole enzyme that adds an O-GlcNAc-modification onto proteins, plays a critical role for pancreatic β-cell survival and insulin secretion. We hypothesized that β-cell Ogt overexpression would confer protection from β-cell failure in response to metabolic stressors, such as high-fat diet (HFD) and streptozocin (STZ). Here, we generated a β-cell-specific Ogt in overexpressing (βOgtOE) mice, where a significant increase in Ogt protein level and O-GlcNAc-modification of proteins were observed in islets under a normal chow diet. We uncovered that βOgtOE mice show normal peripheral insulin sensitivity and glucose tolerance with a regular chow diet. However, when challenged with an HFD, only female βOgtOE (homozygous) Hz mice developed a mild glucose intolerance, despite increased insulin secretion and normal β-cell mass. While female mice are normally resistant to low-dose STZ treatments, the βOgtOE Hz mice developed hyperglycemia and glucose intolerance post-STZ treatment. Transcriptome analysis between islets with loss or gain of Ogt by RNA sequencing shows common altered pathways involving pro-survival Erk and Akt and inflammatory regulators IL1β and NFkβ. Together, these data show a possible gene dosage effect of Ogt and the importance O-GlcNAc cycling in β-cell survival and function to regulate glucose homeostasis.

## 1. Introduction

Pancreatic β-cells are primary modulators of glucose homeostasis, where their failure to secrete insulin in response to appropriate nutrient cues leads to the development of uncontrolled hyperglycemia or type 2 diabetes (T2D). Integrating nutrient status and extracellular signal to exert proper intracellular response is a critical requirement for β-cell function and maintenance of glucose homeostasis. Protein O-GlcNAcylation is a dynamic post-translational modification which involves the addition of one GlcNAc molecule onto serine or threonine of target proteins localized in the cytosol, nucleus, and mitochondria to regulate their stability, function, and localization [1]. O-GlcNAcylation is regulated by two enzymes, namely O-GlcNAc transferase (Ogt) to add GlcNAc onto target proteins and O-GlcNAcase (Oga) to remove GlcNAc from proteins. Ogt is highly expressed in β-cells and regarded as a potent nutrient-sensor, as its substrate uridine diphosphate-N-acetylglucosamine (UDP-GlcNAc) is derived from the hexosamine biosynthetic pathway. Aberrant O-GlcNAcylation is associated with β-cell failure and diabetic complications [2], and Oga has been identified as a T2D susceptibility gene [3].

Ogt has been shown to be a critical regulator in both the development and function of the pancreas [4,5,6]. Deletion of Ogt in pancreas progenitors leads to improper endocrine cell-mass development [6], whereas deficits in glucagon hormone secretion are observed in Ogt loss α-cells [7]. In pancreatic β-cells, Ogt directly targets critical transcriptional (Pdx1 and NeuroD1), translational factors (eIF4G1), and Ca^2+^ regulators (SERCA2) to regulate insulin biosynthesis and secretion [8,9,10,11]. As such, alterations to islet O-GlcNAcylation are correlated with adaptive hyperinsulinemia in both obese mice and humans [11]. Additionally, deletion of Ogt is associated with ER-stress-mediated β-cell survival, leading to severe hyperglycemia and overt diabetes young-adult mice [5]. While these loss-of-function studies and models of hypo-O-GlcNAcylation revealed the requirement of Ogt in β-cell function and increased Ogt expression, a model of hyper-O-GlcNAcylation has not been tested in β-cells.

The present study investigated the role of Ogt overexpression in regulating β-cell mass and function in vivo. We report that, while transgene induction was successful in both male and female islets, only the female islets exhibited a higher Ogt protein level and O-GlcNAcylation level. These transgenic mice maintained normal glucose homeostasis under a normal chow diet. However, when challenged to diabetogenic conditions, the mice developed glucose intolerance with lower fasting serum insulin levels. While female mice are normally resistant to low-dose STZ assault, we found that the female transgenic mice developed severe hyperglycemia and glucose intolerance within weeks of STZ treatment. Altogether, these data implicate that Ogt gain-of-function is as detrimental to β-cell health and function as Ogt loss-of-function, and they further highlight that the proper cycling of this post-translational glycosylation is critical for proper cellular homeostasis.

## 2. Materials and Methods

### 2.1. Mouse Models and Glucose Metabolism Phenotyping

Pancreatic β-cell Ogt overexpression (βOgtOE) is generated by crossing a mouse line expressing cre-recombinase under rat insulin promoter (*rip-cre*, from Dr. Pedro Herrera, University of Geneva) with a mouse line harboring a rat OGT transgene in the *Rosa26* locus (Rosa26-STOPfloxed-Flag-HA-rOGT-IRES-eGFP from Xiaoyong Yang, Yale University). Ogt overexpression has been successfully induced in adipocytes or enteroendocrine L cells, using the Cre-lox system [12,13]. All mice were group housed on a 14:10 light–dark cycle. A high-fat diet (HFD) (60% kcal of fat, D12492) was purchased from Research Diets. Low-dose streptozocin (50 mg/kg; Sigma, St. Louis, MO, USA) was injected for 5 days, consecutively, and followed up for 4 weeks to assess blood glucose, glucose tolerance, and serum insulin levels. Mice were fasted for 16 or 6 h prior to the test for IP glucose tolerance (IPGTT, glucose 2 g/kg of body weight) or insulin tolerance (ITT, 0.75 Unit of insulin/kg of body weight, Humalog, Eli Lilly), respectively.

### 2.2. Primary Mouse Islets Isolation and In Vitro Glucose-Stimulated Insulin Secretion

Islet isolation and in vitro glucose-stimulated insulin secretion were performed as described [14]. In brief, perfusion of collagenase solution (1 mg/mL) is carried out through common bile duct puncture, and inflated pancreas is collected and digested in 37 °C. Homogenate is filtered by using 70 µm cell strainer. Islets are then hand-picked and cultured overnight in 37 °C, in an incubator, with RPMI media containing 5 mM glucose, 10% FBS, and 1% P/S, prior to collection or in vitro testing.

### 2.3. Western Blotting

Islets were lysed in 1xRIPA buffer containing protease inhibitor, phosphatase inhibitor, and 1% SDS. Lysate was sonicated briefly, and the supernatant was used to measure protein concentration by BCA assay. Equal amount of protein was loaded onto SDS–gel and transferred into PVDF membrane. The blot was blocked with 10% milk and incubated in primary antibody, at 4 °C, overnight. It was followed by secondary incubation with appropriate secondary antibody and subsequent imaging, using ECL reagents. The following primary antibodies were used: pan-O-GlcNAc (RL2) from Abcam; OGT, GFP, tubulin, and actin from Cell Signaling; and Pdx1 from Millipore.

### 2.4. Immunostaining

Pancreata fixed in 3.7% formaldehyde were embedded in paraffin and sectioned into 5 um–thick slices for every 200 μm through the depth of pancreas. Minimum of 5 representative tissue sections from 5 different region of pancreas were used for cell-mass analysis. Sections were deparaffinized; treated with citrate buffer (10 mM Sodium Citrate, 0.05% Tween-20) for antigen retrieval; and then incubated at 4 °C with primary antibody against insulin (Dako), glucagon, and RL2 (Pan-O-GlcNAc antibody) (Abcam, Waltham, MA, USA). Following overnight incubation in 4 °C, the sections were stained with secondary antibodies conjugated to FITC or Cy5 (Jackson Immunoresearch) and processed with DAPI solution. All images were taken on motorized microscope (ECLIPSE NI-E; Nikon, Melville, NY, USA). For β-cell and α-cell mass calculation, the ratio of insulin-positive area (β-cell) or glucagon-positive area (α-cell) over total pancreas area, assessed from FJII software (1.52p; National Institute of Health, USA), was multiplied by the pancreas weight. TUNEL staining was performed by using Millipore ApopTag Red in situ Apoptosis Detection kit, following manufacturer’s protocol.

### 2.5. RNA Isolation, qPCR and RNAseq Analysis

RNA was isolated from islets, using RNeasy plus micro kit, following manufacturer’s instructions. For qPCR, cDNA was synthesized from islet RNA with high-capacity cDNA reverse-transcription kit (Applied Biosystems, Waltham, MA, USA). Relative gene expression was assessed by using Sybr Green (Applied Biosystems) on QuantStudio 6 Flex Real-Time PCR systems and calculated with ΔΔ cycle threshold (ΔΔCT) normalized to loading control. Primer sequences are listed in Supplementary Materials Table S1. For RNA sequencing, DNase treatment was included in the RNA isolation, and integrity (>8) was validated by using an Agilent 2200 TapeStation. During sequencing, 125 bp FastQ paired-end reads (*n* = 15.6 million per sample) were trimmed using Trimmomatic (v 0.33; Potsdam, Germany) enabled with the optional “-q” option; 3 bp sliding-window trimming from 3′ end requiring minimum Q30. Quality-control checks on raw sequence data for each sample were performed with FastQC. Read mapping was performed via Hisat2 (v2.0.2; Dallas, TX & Baltimore, MD, USA), using the UCSC mouse genome (mm10) as reference. Gene quantification was performed via Feature Counts for raw read counts. Differentially expressed genes were identified by using the edgeR (negative binomial) feature in CLC Genomics Workbench, using raw read counts. The generated list was filtered based on a minimum 1.5X absolute fold-change and *p* < 0.05. Canonical pathways, upstream regulator, and network analysis were performed by using Ingenuity Pathway Analysis. The RNAseq dataset from β-cell OGT knockout islets (combined) from our previous work [11] was compared to the current RNAseq dataset. The overlap was assessed and diagrammed by using Venny 2.1 (https://bioinfogp.cnb.csic.es/tools/venny/ accessed on 18 September 2021).

### 2.6. Statistical Analysis

Data are presented as mean ± SEM and were analyzed by using unpaired two-tailed Student’s *t*-tests. Multiple outcome data were assessed by using repeated measures 2-way ANOVA. Statistical analyses were performed in GraphPad Prism version 7, with a significance threshold of *p* < 0.05.

## 3. Results

### 3.1. Transgenic Ogt Overexpression Female Mice Specific Upregulation of Ogt in Pancreatic Islets

**Figure 1:**
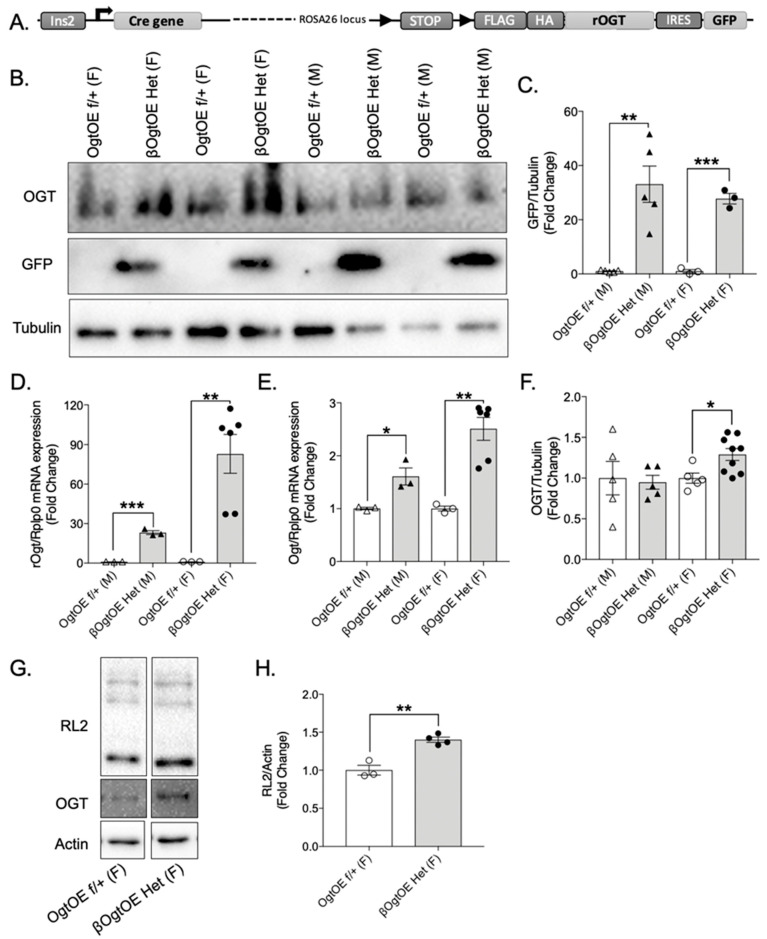
Validation of Ogt overexpression in Pancreatic Islets. (**A**) Diagram showing the genetic scheme of mice carrying Ins2 driven Cre recombinase and ROSA26-STOP^flox^-rOgt/GFP in ROSA26 locus. (**B**) Representative Western blots of Ogt, GFP, and tubulin from pancreatic islets from 4–10-week-old OgtOE f/+ control and βOgtOE Het mice with quantification; fold-change by each sex (*n* = 5 males, 3–9 females) (**C**,**F**). mRNA analysis of transgenic rOgt (**D**) and Ogt (**E**) from OgtOE f/+ and βOgtOE Het islets; fold-change by each sex (*n* = 3 males, 3–6 females). (**G**) Islets from female control and transgenic animals were incubated in low glucose (2.5 mM) for 6 h and immunoblotted against RL2, pan-O-GlcNAc antibody (**H**) and actin (*n* = 3–4). Fold-change to control. Statistical analyses were conducted by using unpaired two-tailed Student’s *t*-test, with significance * *p* < 0.05, ** < 0.01, *** < 0.001.

To investigate the consequences of Ogt overexpression in β-cells, transgenic mice were generated by crossing animals harboring the Rosa26-STOP^flox^-Ogt^OE^ and Ins2 promoter driven Cre-recombinase (Figure 1A). Expression of the reporter gene, GFP, was present only in islets of βOgtOE mice, thus confirming the efficiency the Cre transgene (Figure 1B,C). Assessment of mRNA levels of the exogenous transgenic Ogt (rOgt) showed a significant increase in both male and female islets from βOgtOE, as compared to their respective control floxed mice (Figure 1D). Consistently, Ogt transcript levels were significantly increased in the transgenic islets (Figure 1E). Although the islets of male transgenic mice expressed the reporter GFP, as well as increased Ogt transcript, Ogt protein levels were not altered when compared to the controls (Figure 1F). A significant increase in Ogt protein levels was only obtained in islets of female βOgtOE mice (Figure 1F). Further analysis of female islets revealed a significant increase in total protein O-GlcNAcylation levels (assessed by pan-O-GlcNAc antibody, RL2) in the transgenic islets, suggesting increased Ogt activity (Figure 1G,H). Moving forward, glucose homeostasis phenotyping was performed in both heterozygous (βOgtOE Het) and homozygous (βOgtOE Hz) lines, with their respective littermate controls in both sexes.

### 3.2. Male and Female βOgtOE Het or βOgtOE Hz Mice Overexpressing Ogt Exhibit Normal Glucose Homeostasis under Normal Chow Diet

**Figure 2:**
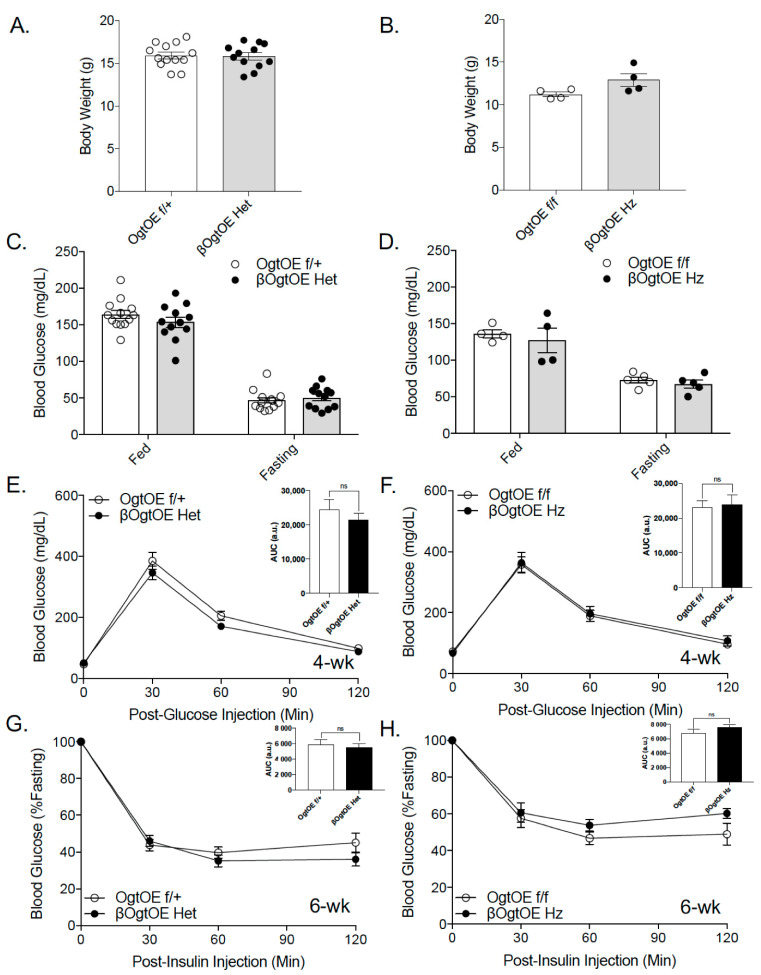
Ogt overexpression in β-cells does not alter glucose homeostasis under normal chow diet. Body weight from female βOgtOE Het (**A**) and βOgtOE Hz (**B**) with their respective littermate controls at 4 weeks of age. Random blood glucose under random fed and post-16 h fast from female βOgtOE Het (**C**) and βOgtOE Hz (**D**) with their respective littermate controls at 4 weeks of age (*n* = 12–13 βOgtOE Het, *n* = 4 βOgtOE Hz). Intraperitoneal glucose tolerance test (glucose 2 g/kg body weight) from βOgtOE Het (**E**) and βOgtOE Hz (**F**), with littermate controls at 4 weeks of age (*n* = 10–13 βOgtOE Het, *n* = 5 βOgtOE Hz). Intraperitoneal insulin tolerance test (insulin 0.75 U/kg) from βOgtOE Het (**G**) and βOgtOE Hz (**H**), with littermate controls at 6 weeks of age (*n* = 9–10 βOgtOE Het, *n* = 5 βOgtOE Hz). Area under the curve (AUC) of each graph is shown as inset. Statistical analyses were conducted by using unpaired two-tailed Student’s *t*-test or 2-way ANOVA, with significance *p* < 0.05.

First, metabolic phenotypes were assessed under a normal chow diet to assess the immediate effects of Ogt overexpression in β-cells. No alterations in body weight and random blood glucose in 4–10-week-old female βOgtOE Het or βOgtOE Hz were detected (Figure 2A–D). Next, glucose homeostasis was assessed with intraperitoneal glucose tolerance test (IPGTT) and insulin tolerance test (ITT) in transgenic mice at 4 weeks and 6 weeks of age, respectively. There were no notable differences observed in both glucose tolerance and insulin sensitivity of female βOgtOE Het or βOgtOE Hz mice compared to their littermate controls (Figure 2E–H). Male βOgtOE Het or βOgtOE Hz mice, which showed no alterations to the islet Ogt level, exhibited no differences in any metabolic parameters tested (Supplementary Figure S1). Together, these data show that there were no metabolic consequences to β-cell Ogt overexpression under a normal chow diet.

### 3.3. Female βOgtOE Hz Mice Overexpressing Ogt Showed Increased Susceptibility to Glucose Intolerance under High-Fat Diet Challenge

**Figure 3:**
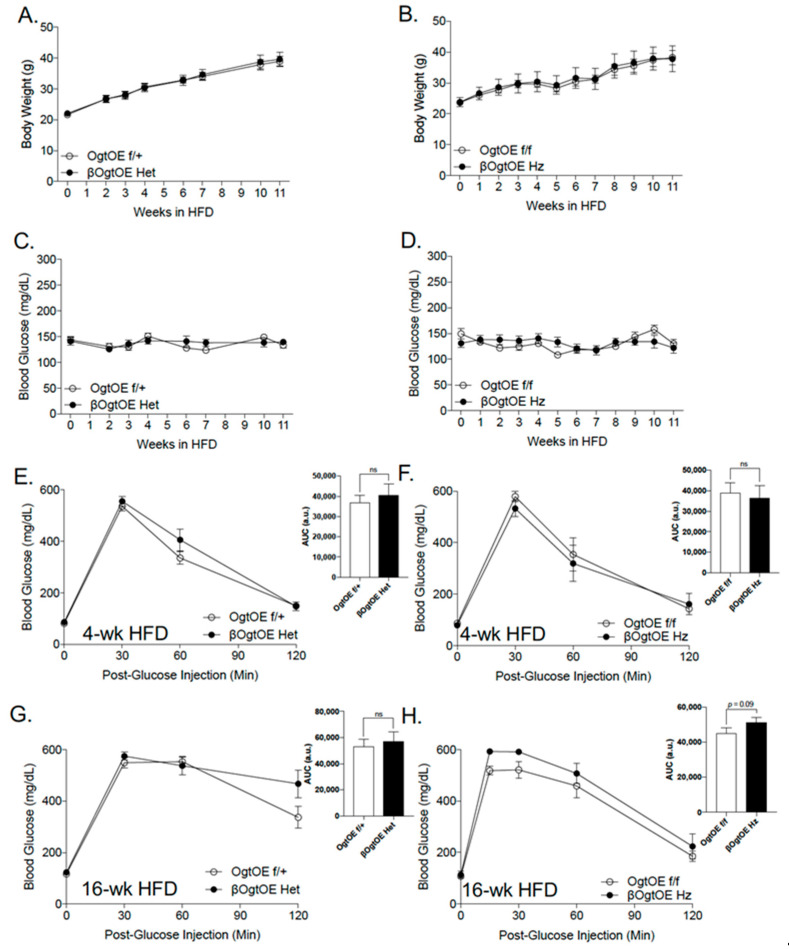
Chronic high-fat diet leads to impaired glucose tolerance in mice harboring OGT overexpressing β-cells. Body weight over the course of 11 weeks of HFD in female βOgtOE Het (**A**) and βOgtOE Hz (**B**) with respective littermate controls (*n* = 13–14 βOgtOE Het, *n* = 5 βOgtOE Hz). Random blood glucose over the course of 11 weeks of HFD in βOgtOE Het (**C**) and βOgtOE Hz (**D**) with respective littermate controls (*n* = 13–14 βOgtOE Het, *n* = 5 βOgtOE Hz). Intraperitoneal glucose tolerance test (glucose 2 g/kg body weight) from βOgtOE Het and βOgtOE Hz, with littermate controls at 4 weeks of HFD (**E**,**F**) and 16 weeks of HFD (**G**,**H**) (*n* = 10–13 βOgtOE Het, *n* = 7–8 βOgtOE Hz). Area under the curve (AUC) of each graph is shown as inset. Statistical analyses were conducted by using 2-way ANOVA with multiple comparisons and two-tailed Student’s *t*-test, with significance *p* < 0.05. ns = not significant.

Ogt has been shown to play a critical role in mounting the appropriate β-cell adaptation to a hyper-nutrient environment [11]. To understand the physiologic response to metabolic stress with β-cell Ogt overexpression, the mice were fed a high-fat diet (HFD, 60% Kcal), starting at the age of 8–14 weeks. As expected, all animals gained body weight whilst maintaining euglycemia over 11 weeks of HFD, and no difference between transgenic mice (Het or Hz) and controls was observed (Figure 3A–D). Female transgenic mice (Het and Hz cohorts) appeared to have similar glucose homeostasis compared to the control mice at 4 weeks post-HFD (Figure 3E,F). At 16 weeks post-HFD, βOgtOE Het animals showed normal glucose tolerance, but they showed a higher glucose level at 2-h timepoint post-bolus glucose i.p. injection (Figure 3G). However, βOgtOE Hz mice developed a mild glucose intolerance, and AUC *p*-value = 0.09 (Figure 3H).

We then assessed Ogt, Oga and RL2 levels post 20-weeks of HFD treatment (mice were non-fasted) to see the status of these proteins. By immunofluorescence imaging, we observed increase in O-GlcNAcylation (by staining with RL2 antibody, Supplementary Figure S2A) in insulin-positive β-cells from WT in NCD and WT control mice fed an HFD, compared to the control, with further increase in the βOgtOE Het animals. However, via the analysis of whole islets (non-purified β-cells) by Western blotting, a non-significant change in the level of Ogt, Oga, and O-GlcNAcylation (RL2) was observed in both the βOgtOE Het and βOgtOE Hz (Supplementary Figures S2B and S3A–D). The unaltered level of Ogt can be explained in part by the presence of non-β-cells in total islet lysates, non-fasted status of the animals, downregulation of Ogt protein level after 18 weeks of HFD treatment in female mice [11], or post-transcription regulation and stability of Ogt. As in normal chow, the male control and transgenic mice showed no differences in their metabolic responses to HFD (Supplementary Figure S4).

**Figure 4:**
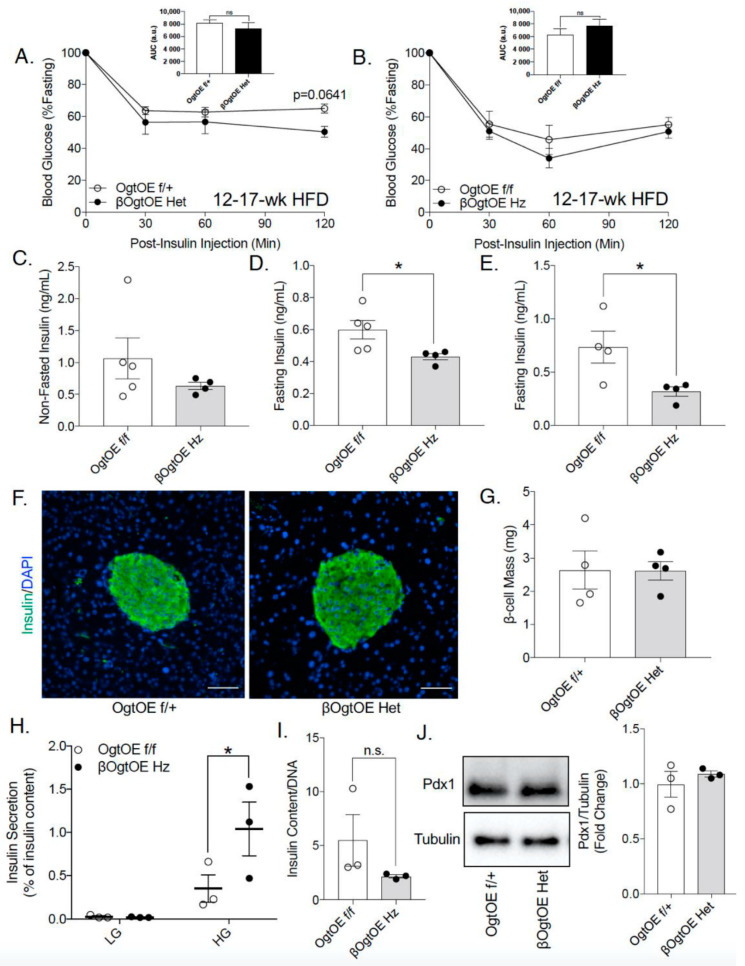
β-cell OGT overexpressing mice exhibits lower circulating insulin under high-fat diet. Intraperitoneal insulin tolerance test (insulin 0.75 U/kg) from female βOgtOE Het (**A**) and βOgtOE Hz (**B**), with littermate controls at 12–17 weeks of HFD (*n* = 9–10 βOgtOE Het, *n* = 5 βOgtOE Hz). Fed insulin at 10 weeks of HFD (**C**) and 16-h fasted insulin at 10 weeks (**D**) and 18 weeks of HFD (**E**) from βOgtOE Hz and littermate control mice (*n* = 4–5). Representative images of β-cells at 20× magnification (**F**) and β-cell mass analysis (**G**) from βOgtOE Het and littermate control mice at 20–25 weeks of HFD (*n* = 5). Scale Bar = 50 μm. In vitro insulin secretion in response to low glucose (LG, 3 mM) to high glucose (HG, 16.7 mM) in βOgtOE Hz and control islets (**H**); normalized to insulin content (*n* = 3). Post-GSIS insulin content normalized to islet DNA (**I**) (*n* = 3). Pdx1 protein level normalized to tubulin expression from βOgtOE Het and control islets (**J**) (*n* = 3). Area under the curve (AUC) of each graph is shown as inset. Statistical analyses were conducted by using unpaired two-tailed Student’s *t*-test or 2-way ANOVA with multiple comparison; significance * *p* < 0.05. ns = not significant.

The female transgenic mice (βOgtOE Hz and βOgtOE Het) exhibited normal insulin sensitivity (Figure 4A,B), suggesting that the mild impairment in glucose tolerance under HFD could be due to potential defects in either pancreatic β-cell mass or function. βOgtOE Hz transgenic mice showed no alterations in non-fasted serum insulin levels (Figure 4C). In contrast, there was a decrease in serum insulin post-16-h fasting at 10- and 18-week HFD in βOgtOE Hz mice (Figure 4D,E). β-cell mass analysis revealed no significant changes in the βOgtOE Het compared to controls in HFD (Figure 4F,G). In vitro glucose stimulated insulin secretion (GSIS, static incubation) in βOgtOE Hz islets showed increase response to glucose (Figure 4H). GSIS of βOgtOE Het islets showed a similar response relative to control (Supplementary Figure S5). Total insulin content and Pdx1 protein, one of the critical regulators of insulin biosynthesis, were comparable between βOgtOE Het and control animals (Figure 4I,J).

### 3.4. OgtOE Hz Mice Shows Increased Susceptibility to Streptozocin (STZ)-Induced Hyperglycemia

**Figure 5:**
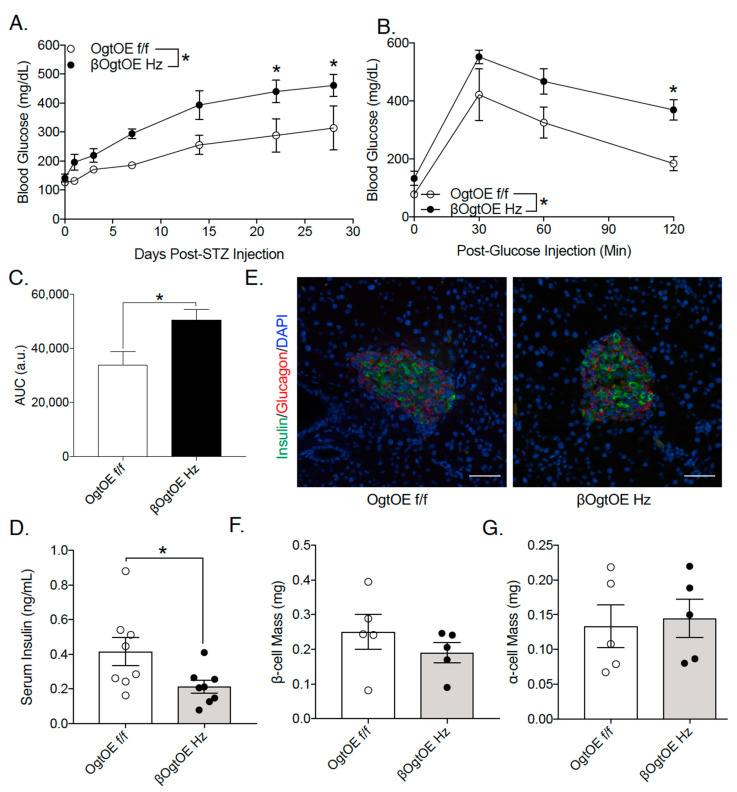
OGT overexpression sensitizes female β-cells to diabetogenic streptozocin treatment. Blood glucose over 4 weeks post-STZ injection in female βOgtOE Hz (**A**) with littermate controls (*n* = 5). Intraperitoneal glucose tolerance test (glucose 2 g/kg body weight) (**B**,**C**) (*n* = 3–4) and circulating insulin levels after 16-h fasting (**D**) in female βOgtOE Hz and control mice 4 weeks post-STZ (*n* = 8). Representative images of islets showing insulin, glucagon, and DAPI; imaged at 20× (**E**). Scale Bar = 50μm. β-cell (**F**) and α-cell mass (**G**) analysis from 4 weeks post-STZ in βOgtOE Hz and littermate controls (*n* = 5). STZ was induced in 8–10-week-old mice. Statistical analyses were conducted by using unpaired two-tailed Student’s *t*-test or 2-way ANOVA with multiple comparisons; significance * *p* < 0.05.

Previous studies have implicated the possible role of glucosamine metabolism in STZ-induced β-cell apoptosis [15]. STZ treatment blocks Oga, and thus increases O-GlcNAcylation in β-cells [16,17,18]. We hypothesize that increased Ogt levels may predispose the βOgtOE mice to diabetes. Hence, 8–10-week-old transgenic mice, along with respective controls, were subjected to low-dose STZ injection for 5 days. STZ treatment in male βOgtOE Hz or Het and control mice led to the development of comparable level of hyperglycemia, validating the efficacy of STZ (Supplementary Figure S6A,B). As expected in female control group, the mice were relatively resistant to low-dose STZ treatment, showing non-significant changes in glucose levels post-STZ injection (Figure 5A; Supplementary Figure S6C). βOgtOE Het female mice also exhibited non-significant changes in blood glucose or glucose tolerance with STZ treatment (Supplementary Figure S6C,D); however, βOgtOE Hz female mice showed increased susceptibility to STZ and developed significant hyperglycemia relative to controls (Figure 5A); these suggest the possible dosage effect of Ogt. Consistently, βOgtOE Hz mice became glucose intolerant and showed lower fasting insulin levels (Figure 5B–D). Consequences to STZ treatment include the reduction of functional β-cell mass and increase in α-cell mass [19]. While there was a trend towards higher apoptosis by TUNEL analysis in βOgtOE Hz β-cells (Supplementary Figure S6E), βOgtOE Hz showed no gross differences in their β- and α-cell mass 26 days post-STZ treatment, compared to their littermate controls (Figure 5D–F).

### 3.5. Altered Transcriptomics in βOgtOE Hz Islets

**Figure 6:**
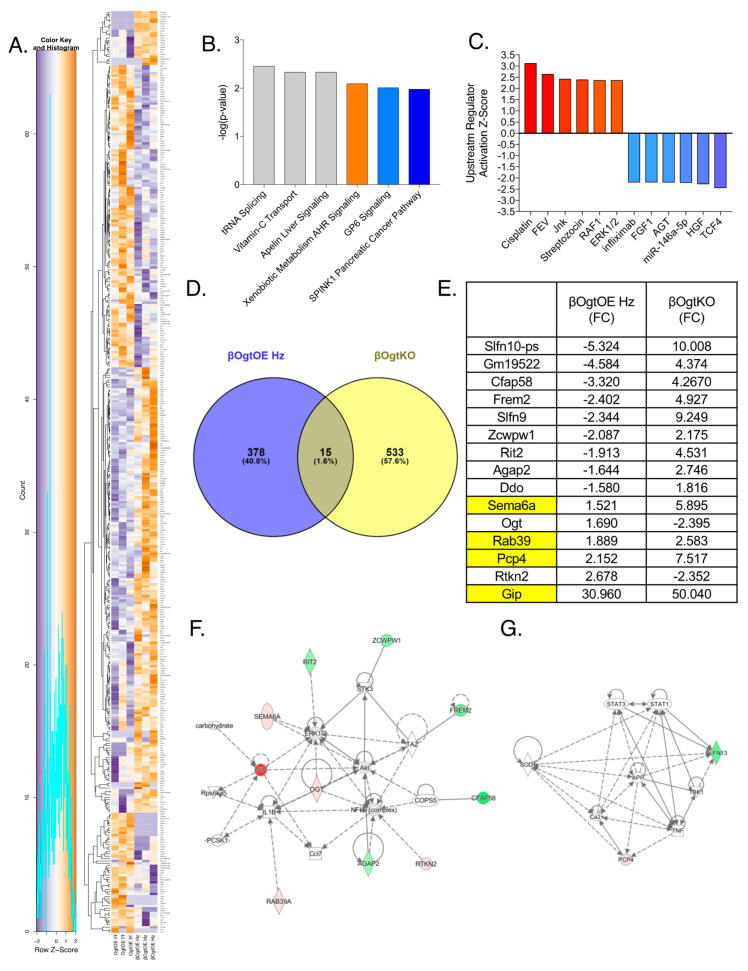
Transcriptomic analysis reveals differentially expressed genes in β-cell OGT overexpressing islets. In-depth RNA sequencing of control and βOgtOE Hz female islets (50 days old; *n* = 3 identified 393 differentially expressed genes (DEGs) (1.5–fold minimum change; *p* < 0.05). Heatmap (**A**), top 6 altered canonical pathways (**B**), and activated and inhibited upstream regulators in line with IPA-predicted activation status (**C**). Venn diagram of DEGs from βOgtOE Hz vs. βOgtKO female islets for overlapping genes of interest (**D**) with fold-change (FC) in each group presented (**E**). In (**E**), yellow highlight shows the genes that are altered in the same direction in both βOgtOE Hz and βOgtKO RNAseq analysis. Network analysis of the overlapping genes of interest from βOgtOE Hz dataset, involving 10 (**F**) or 2 (**G**) sets of genes; red = upregulated; green = downregulated.

To assess potential molecular regulators of β-cell function downstream of Ogt, we performed RNA deep sequencing on primary islets from female βOgtOE Hz islets and littermate control mice and identified 393 differentially expressed genes (DEGs) (minimum fold-change of 1.5; *p* < 0.05. In Figure 6A, the top ten increased/decreased DEGs are shown in Supplementary Figure S7A). The mRNA of Ogt was significantly increased (1.69-fold-change, Figure 6E) validating overexpression of Ogt in βOgtOE Hz islets. Ingenuity Pathway Analysis (IPA) was used to produce top pathways and predicted upstream regulators of the network of DEGs. The top pathways suggested tRNA splicing, vitamin C transport, apelin liver signaling, metabolism of aryl hydrocarbon receptor (AHR) signaling, glycoprotein VI platelet (GP6) signaling, and SPINK1 pancreatic cancer pathway (Figure 6B). The top-predicted upstream regulators in line with directionality of activation state include activated pathways associated with anticancer cisplatin, MAPK pathways (Jnk, Raf1, and Erk1/2), FEV Transcription Factor, and STZ. 

Top-predicted upstream regulators that may be inhibitory included transcription factor 4 (TCF4), hepatocyte growth factor (HGF), anti-inflammatory miR-146a, angiotensinogen (AGT), fibroblast growth factor (FGF1), and immuno-suppressant infliximab (Figure 6C). Our previous work in RNAseq from female β-cell specific Ogt deleted islets (βOgtKO) revealed 548 DEGs [11]. By comparing these datasets, we identified 15 genes that are commonly altered by gain-of-function and loss-of-function Ogt (Figure 6D). Eleven of these genes showed differential regulation between the two models, where the expression was reduced in βOgtOE Hz but increased in βOgtKO islets, while four of the genes showed increased expression in both transgenic models (Figure 6E). Using IPA, the network generated from these genes shows Ogt interactions with pro-survival pathway Erk1/2 and Akt, and inflammatory regulatory proteins IL1β and NF-kβ (Figure 6F for βOgtOE Hz, and Supplementary Figure S7B for βOgtKO network). IL1β mRNA was increased in islets of βOgtOE Hz (1.95, *p* = 0.038). Interestingly, Slfn10, belonging to the Schlafen family proteins involve in cell proliferation and induction of immune responses, was highly decreased in βOgtOE Hz but increased in βOgtKO islets (Figure 6E,G).

## 4. Discussion

Chronic hyperglycemia and hyperlipidemia are associated with increased O-GlcNAcylation [20]. However, the metabolic consequences of enhanced O-GlcNAcylation have not been explored previously in the pancreas. In this study, we show that mice with β-cell-specific Ogt overexpression in a model of hyper-O-GlcNAcylation exhibit normal glucose homeostasis under a normal chow diet, but they display glucose intolerance under diabetogenic conditions, such as HFD or STZ treatment.

Ogt loss in β-cells causes severe β-cell failure and overt-diabetes in young adult mice [5] by impacting multiple pathways critical for β-cell health and function. Whole-body haploinsufficiency of Oga in rodents increases insulin secretion in females in both normal chow and HFD [21]. Thus, we had hypothesized that enhanced β-cell O-GlcNAcylation by β-Ogt overexpression would confer protection from a HFD challenge; however, female transgenic βOgt overexpressor (Hz) mice developed mild glucose intolerance with reduced fasting serum insulin. At the islet level, increased insulin secretion was observed in βOgtOE Hz, as is consistent with the idea that Ogt promotes β-cell function [5]. However, βOgtOE Hz mice display reduced serum insulin levels that is not associated with β-cell mass deficiency; therefore, additional studies are needed, such as dynamic insulin secretion perifusion analysis vs. static incubation. Consistently, mice with partial and mosaic deletion of β-cell Ogt under HFD also develop hypoinsulinemia without alterations in β-cell mass [11]. The mild hypoinsulinemia in the βOgtOE Hz may have contributed to the trend toward improvement in insulin tolerance, as hyperinsulinemia drives insulin resistance and obesity [22,23]. However, additional gold-standard assays, such as in vivo clamps, are needed to assess the role of βOgtOE Hz in insulin sensitivity.

Similar to the βOgt overexpressor mice, Oga HET animals displayed sexual dimorphic phenotypes. Oga HET females, not Oga HET males, develop obesity and were not protected from diet-induced obesity [21]. Moreover, transgenic mice overexpressing GFAT in adipose tissue also exhibited gender-related differences in metabolic response (i.e., lower glucose disposal rate, lower 2-deoxy-d-glucose uptake, and higher adiponectin levels than males) [24]. In the current study, only female βOgt overexpressor Hz mice displayed glucose intolerance in response to HFD and STZ. The male βOgt overexpressor and control mice were indistinguishable regarding glucose and insulin tolerance under HFD treatment. The sexual dimorphic phenotypes in βOgt overexpressor might be explained in part by lack of Ogt overexpression and increased activity in male islets. It is important to point out that, while both male and female islets showed efficient transgene recombination via GFP reporter gene expression, only female βOgtOE islets achieved a significantly higher protein expression of Ogt, which resulted in higher O-GlcNAcylation. The exogenous Ogt RNA level was also higher in females, potentially suggesting transcriptional or post-transcriptional regulatory differences between male and female transgenic animals. Overexpression of the same Ogt transgene in adipose or enteroendocrine L cells does not show sexual dimorphic expression [12,13]. Future studies are required to elucidate the sex-dependent mechanisms of Ogt transgene overexpression in islets and to explore possible mechanisms of post-transcriptional and stability regulation of Ogt.

In the current study, female βOgtOE Hz mice also developed hyperglycemia post-treatment with the β-cell-specific toxin, STZ. As a GlcNAc analog, STZ causes apoptosis in part by blocking Oga. STZ treatment increases GlcNAc levels in islets, and transgenic animals with impaired glucosamine synthesis are resistant to the diabetogenic effect of STZ [15], suggesting a link between glucosamine metabolism and STZ toxicity. Female mice, which have shown to be resistant to low-dose treatments of STZ, became sensitized with Ogt overexpression. It is possible that, with STZ treatment, there is a prolong accumulation of *O*-GlcNAc on critical substrates that are involved in the apoptotic response. This is supported by increased apoptosis in β-cells of female βOgtOE Hz mice. Not only does STZ increase *O*-GlcNAc levels, but it also increases Ogt expression [25], which can further exacerbate chronic *O*-GlcNAcylation of target proteins, which perturbs *O*-GlcNAc cycling [26]. It is interesting that the βOgtOE Het mice presented similar levels of glucose as control in response to STZ treatment, suggesting that there might be an Ogt dosage effect in the female overexpressing Ogt (Het vs. Hz), and an interest to pursue further in the future. Indeed, female mice lacking two alleles of Ogt in their β-cells develop hyperglycemia and diabetes at six months of age [5], whereas mice lacking one allele ablation of Ogt show normal glucose tolerance at the same time point [11].

While the mechanisms of insulin deficit in Ogt loss model included several pathways, such as increased ER stress, insulin biogenesis and processing dysfunction, and reduced pro-survival signaling networks [5,6,10,11], the transcriptome analysis revealed a small number of overlapping DEGs between islets with loss or gain of Ogt. This is not surprising, given the drastic differences in the metabolic health and β-cell mass status between the OgtKO (β-cell mass failure and overt-diabetes) [5] vs. OgtOE (normal β-cell mass and non-diabetic) mice. Key pathways that remained altered in both models include the MAPK and Akt signaling pathways, both of which have been shown to regulate β-cell function [27,28] and β-cell mass [29]. The association between Ogt and NF-Kβ network was intriguing, and Ogt-mediated O-GlcNAcylation has been shown to promote NF-Kβ activation and inflammation in acute pancreatitis [30]. The possible role of Ogt in inflammatory response in β-cell is unexplored, but Ogt has been implicated to suppress macrophages inflammation activation [31,32].

## 5. Conclusions

In summary, an adequate level of Ogt and O-GlcNAc cycling are required for appropriate pancreatic β-cell function and glucose homeostasis. Loss of Ogt leads to the many hallmarks of β-cell failure and overt-diabetes in a normal chow diet. This is quite distinct from the null metabolic benefits of βOgt overexpression in adult mice under a normal chow diet, and adverse outcome in diabetogenic conditions. Taken together, our findings suggest that excessive Ogt expression in β-cells is maladaptive for glucose homeostasis in diabetogenic conditions and highlights the importance of O-GlcNAc cycling and the non-enzymatic functions of Ogt.

## Data Availability

Data available upon request.

## Supplementary Materials

The following are available online at https://www.mdpi.com/article/10.3390/cells10102801/s1. Figures S1–S5: Glucose homeostasis phenotypes in NCD (S1), HFD (S2), insulin secretion (S3), STZ (S4), and RNAseq of OgtKO islets (S5). Figure S6: Blood glucose over 4-wks post-STZ injection in male βOgtOE Het; Figure S7: (A) Top 10 upregulated and downregulated genes from βOgtOE Hz RNAseq. (B) Net-work analysis of the overlapping genes of interest from βOgtKO dataset, involving 10 (network 1) or 2 (network 2) sets of genes; Table S1: Primer sequences list.

## References

[1] Bond M.R., Hanover J.A. (2015). A little sugar goes a long way: The cell biology of O-GlcNAc. J Cell Biol..

[2] Issad T., Masson E., Pagesy P. (2010). O-GlcNAc modification, insulin signaling and diabetic complications. Diabetes Metab..

[3] Lehman D.M., Fu D.J., Freeman A.B., Hunt K.J., Leach R.J., Johnson-Pais T., Hamlington J., Dyer T.D., Arya R., Abboud H. (2005). A single nucleotide polymorphism in MGEA5 encoding O-GlcNAc-selective N-acetyl-beta-D glucosaminidase is associated with type 2 diabetes in Mexican Americans. Diabetes.

[4] Lubas W.A., Frank D.W., Krause M., Hanover J.A. (1997). O-Linked GlcNAc transferase is a conserved nucleocytoplasmic protein containing tetratricopeptide repeats. J. Biol. Chem..

[5] Alejandro E.U., Bozadjieva N., Kumusoglu D., Abdulhamid S., Levine H., Haataja L., Vadrevu S., Satin L.S., Arvan P., Bernal-Mizrachi E. (2015). Disruption of O-linked N-Acetylglucosamine Signaling Induces ER Stress and beta Cell Failure. Cell Rep..

[6] Baumann D., Wong A., Akhaphong B., Jo S., Pritchard S., Mohan R., Chung G., Zhang Y., Alejandro E.U. (2020). Role of nutrient-driven O-GlcNAc-posttranslational modification in pancreatic exocrine and endocrine islet development. Development.

[7] Essawy A., Jo S., Beetch M., Lockridge A., Gustafson E., Alejandro E.U. (2021). O-linked N-Acetylglucosamine Transferase (OGT) regulates pancreatic alpha-cell function in mice. J. Biol. Chem..

[8] Gao Y., Miyazaki J., Hart G.W. (2003). The transcription factor PDX-1 is post-translationally modified by O-linked N-acetylglucosamine and this modification is correlated with its DNA binding activity and insulin secretion in min6 beta-cells. Arch. Biochem. Biophys..

[9] Andrali S.S., Qian Q., Ozcan S. (2007). Glucose mediates the translocation of NeuroD1 by O-linked glycosylation. J. Biol. Chem..

[10] Jo S., Lockridge A., Alejandro E.U. (2019). eIF4G1 and carboxypeptidase E axis dysregulation in O-GlcNAc transferase-deficient pancreatic beta cells contributes to hyperproinsulinemia in mice. J. Biol. Chem..

[11] Lockridge A., Jo S., Gustafson E., Damberg N., Mohan R., Olson M., Abrahante J.E., Alejandro E.U. (2020). Islet O-GlcNAcylation Is Required for Lipid Potentiation of Insulin Secretion through SERCA2. Cell Rep..

[12] Yang Y., Fu M., Li M.D., Zhang K., Zhang B., Wang S., Liu Y., Ni W., Ong Q., Mi J. (2020). O-GlcNAc transferase inhibits visceral fat lipolysis and promotes diet-induced obesity. Nat. Commun..

[13] Zhao M., Ren K., Xiong X., Cheng M., Zhang Z., Huang Z., Han X., Yang X., Alejandro E.U., Ruan H.B. (2020). Protein O-GlcNAc Modification Links Dietary and Gut Microbial Cues to the Differentiation of Enteroendocrine L Cells. Cell Rep..

[14] Lockridge A.D., Baumann D.C., Akhaphong B., Abrenica A., Miller R.F., Alejandro E.U. (2016). Serine racemase is expressed in islets and contributes to the regulation of glucose homeostasis. Islets.

[15] Liu K., Paterson A.J., Chin E., Kudlow J.E. (2000). Glucose stimulates protein modification by O-linked GlcNAc in pancreatic beta cells: Linkage of O-linked GlcNAc to beta cell death. Proc. Natl. Acad. Sci. USA.

[16] Konrad R.J., Mikolaenko I., Tolar J.F., Liu K., Kudlow J.E. (2001). The potential mechanism of the diabetogenic action of streptozotocin: Inhibition of pancreatic beta-cell O-GlcNAc-selective N-acetyl-beta-D-glucosaminidase. Biochem. J..

[17] Roos M.D., Xie W., Su K., Clark J.A., Yang X., Chin E., Paterson A.J., Kudlow J.E. (1998). Streptozotocin, an analog of N-acetylglucosamine, blocks the removal of O-GlcNAc from intracellular proteins. Proc. Assoc. Am. Physicians.

[18] Pathak S., Dorfmueller H.C., Borodkin V.S., van Aalten D.M. (2008). Chemical dissection of the link between streptozotocin, O-GlcNAc, and pancreatic cell death. Chem. Biol..

[19] Takeda Y., Fujita Y., Honjo J., Yanagimachi T., Sakagami H., Takiyama Y., Makino Y., Abiko A., Kieffer T.J., Haneda M. (2012). Reduction of both beta cell death and alpha cell proliferation by dipeptidyl peptidase-4 inhibition in a streptozotocin-induced model of diabetes in mice. Diabetologia.

[20] Ma J., Hart G.W. (2013). Protein O-GlcNAcylation in diabetes and diabetic complications. Expert Rev. Proteom..

[21] Keembiyehetty C., Love D.C., Harwood K.R., Gavrilova O., Comly M.E., Hanover J.A. (2015). Conditional Knock-out Reveals a Requirement for O-Linked N-Acetylglucosaminase (O-GlcNAcase) in Metabolic Homeostasis. J. Biol. Chem..

[22] Mehran A.E., Templeman N.M., Brigidi G.S., Lim G.E., Chu K.Y., Hu X., Botezelli J.D., Asadi A., Hoffman B.G., Kieffer T.J. (2012). Hyperinsulinemia drives diet-induced obesity independently of brain insulin production. Cell Metab..

[23] Templeman N.M., Flibotte S., Chik J.H., Sinha S., Lim G.E., Foster L.J., Nislow C., Johnson J.D. (2017). Reduced Circulating Insulin Enhances Insulin Sensitivity in Old Mice and Extends Lifespan. Cell Rep..

[24] Cooksey R.C., McClain D.A. (2002). Transgenic mice overexpressing the rate-limiting enzyme for hexosamine synthesis in skeletal muscle or adipose tissue exhibit total body insulin resistance. Ann. N. Y. Acad. Sci..

[25] Akimoto Y., Kreppel L.K., Hirano H., Hart G.W. (2000). Increased O-GlcNAc transferase in pancreas of rats with streptozotocin-induced diabetes. Diabetologia.

[26] Bond M.R., Hanover J.A. (2013). O-GlcNAc Cycling: A Link Between Metabolism and Chronic Disease. Annu. Rev. Nutr..

[27] Alejandro E.U., Lim G.E., Mehran A.E., Hu X., Taghizadeh F., Pelipeychenko D., Baccarini M., Johnson J.D. (2011). Pancreatic beta-cell Raf-1 is required for glucose tolerance, insulin secretion, and insulin 2 transcription. FASEB J..

[28] Bernal-Mizrachi E., Fatrai S., Johnson J.D., Ohsugi M., Otani K., Han Z., Polonsky K.S., Permutt M.A. (2004). Defective insulin secretion and increased susceptibility to experimental diabetes are induced by reduced Akt activity in pancreatic islet beta cells. J. Clin. Investig..

[29] Bernal-Mizrachi E., Wen W., Stahlhut S., Welling C.M., Permutt M.A. (2001). Islet beta cell expression of constitutively active Akt1/PKB alpha induces striking hypertrophy, hyperplasia, and hyperinsulinemia. J. Clin. Investig..

[30] Zhang D., Cai Y., Chen M., Gao L., Shen Y., Huang Z. (2015). OGT-mediated O-GlcNAcylation promotes NF-kappaB activation and inflammation in acute pancreatitis. Inflamm. Res..

[31] Li X., Gong W., Wang H., Li T., Attri K.S., Lewis R.E., Kalil A.C., Bhinderwala F., Powers R., Yin G. (2019). O-GlcNAc Transferase Suppresses Inflammation and Necroptosis by Targeting Receptor-Interacting Serine/Threonine-Protein Kinase 3. Immunity.

[32] Yang Y., Li X., Luan H.H., Zhang B., Zhang K., Nam J.H., Li Z., Fu M., Munk A., Zhang D. (2020). OGT suppresses S6K1-mediated macrophage inflammation and metabolic disturbance. Proc. Natl. Acad. Sci. USA.

