# Supplementary material for: Pancreatic β-Cell O-GlcNAc Transferase Overexpression Increases Susceptibility to Metabolic Stressors in Female Mice
Source: Cells. 2021 Oct 19;10(10):2801. doi: 10.3390/cells10102801 (PMC8534823; doi:10.3390/cells10102801)
Supplement: Supplementary file 1 [file cells-10-02801-s001.zip › cells-1407179 -SM for conversion .pdf]

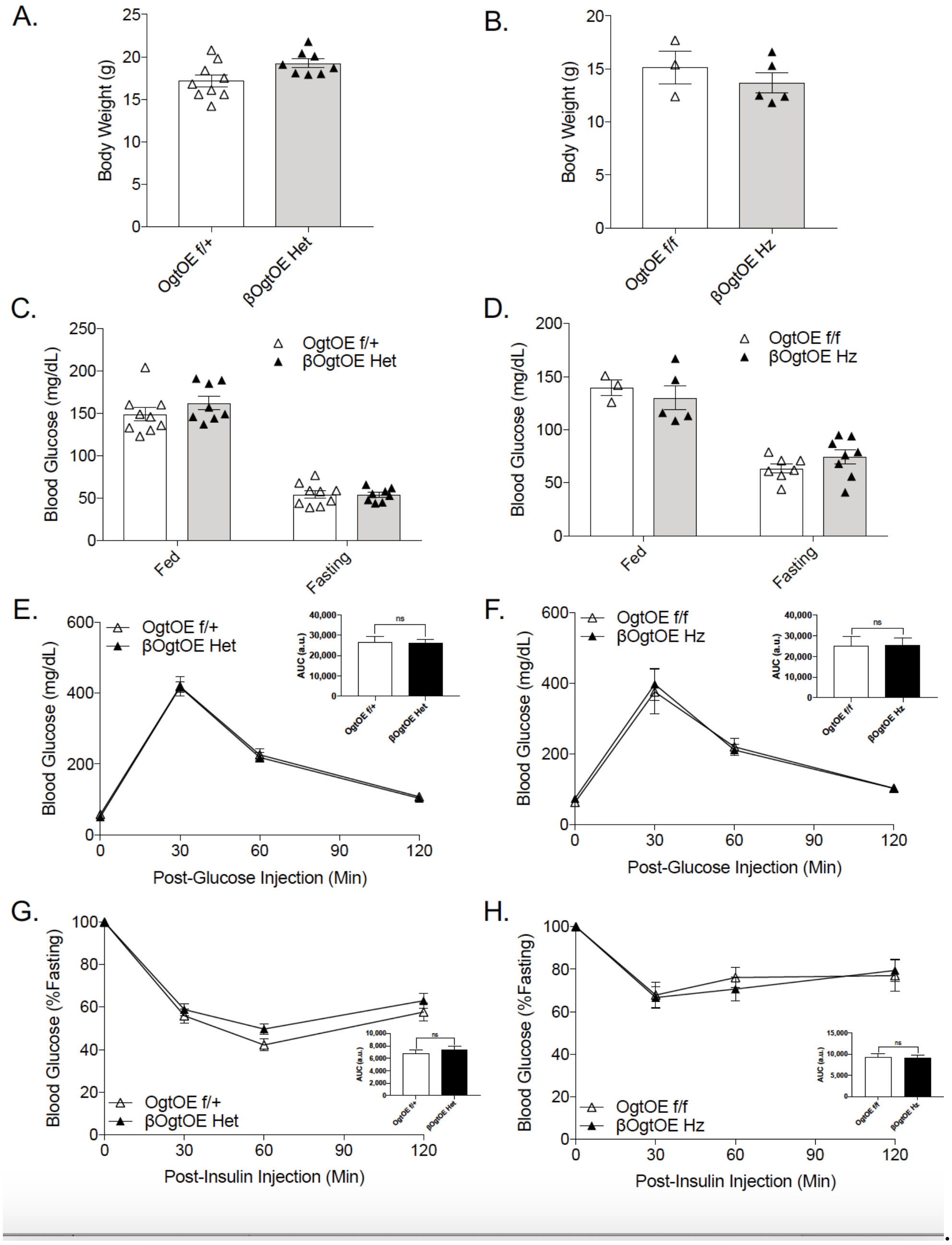

Supplemental Figure S1. Body weight from male βOgtOE Het (**A**) and βOgtOE Hz (**B**) with their respective littermate controls at 4-wks of age age ($n = 3–5$ βOgtOE Het, $n = 8–9$ βOgtOE Hz). Random blood glucose under random fed and post-16 hour fast from βOgtOE Het (**C**) and βOgtOE Hz (**D**) with their respective littermate controls at 4-wks of age ($n = 8–9$ βOgtOE Het, $n = 3–8$ βOgtOE Hz). Intraperitoneal glucose tolerance test (glucose 2 g/kg) from βOgtOE Het (**E**) and βOgtOE Hz (**F**), with littermate controls at 4-wks of age ($n = 8$ βOgtOE Het, $n = 7–8$ βOgtOE Hz). Intraperitoneal insulin tolerance test (insulin 0.75 U/kg) from βOgtOE Het (**G**) and βOgtOE Hz (**H**), with littermate controls at 6-wks of age ($n = 10–14$ βOgtOE Het, $n = 7–8$ βOgtOE Hz). Statistical analyses were conducted using unpaired, two-tailed Student's t test or 2-way ANOVA, with significance $p < 0.05$.

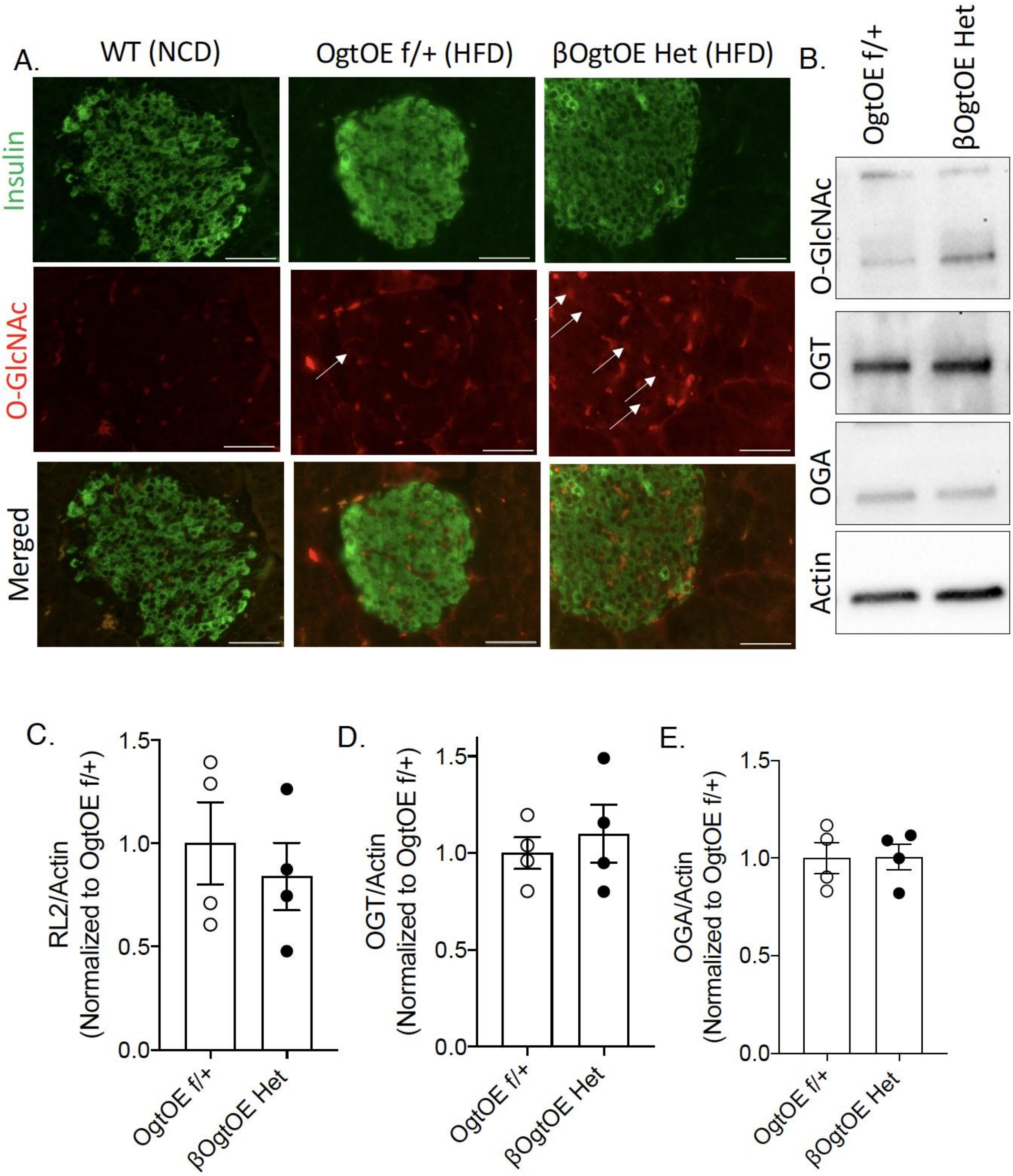

Supplemental Figure S2. (**A**) Representative IF image of WT (normal chow diet; NCD), OgtOE f/+, and βOgtOE Het (high fat diet 20-wks; HFD) pancreas against insulin (green), O-GlcNAcylation (red), and white arrow showing presence of RL2, Table 60. x, and scale Bar = 50μm. (*B*) Representative image of western blot of OgtOE f/+, and βOgtOE Het islets Figure 20. wks, showing pan-O-GlcNAcylation, Ogt, Oga and Actin, *n* = 4.

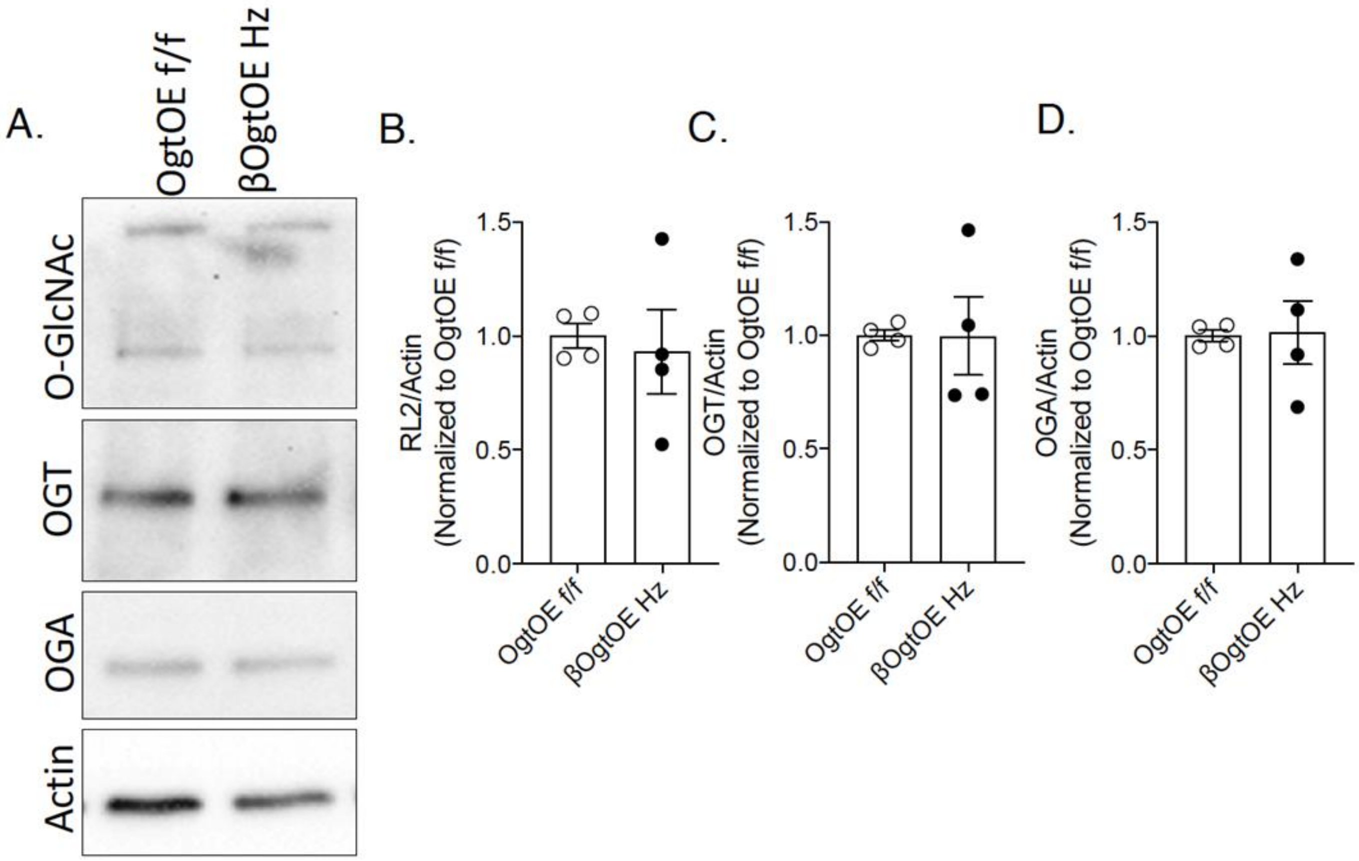

Supplemental Figure S3. (**A**) Representative image of western blot of OgtOE f/f, and βOgtOE Hz islets from mice fed HFD Figure 20. wks, showing pan-O-GlcNAcylation, Ogt, Oga and Actin, n = 4. (**B–D**) are quantifications of RL2/Actin (**B**), Ogt/Actin (**C**), and Oga/Actin (**D**).

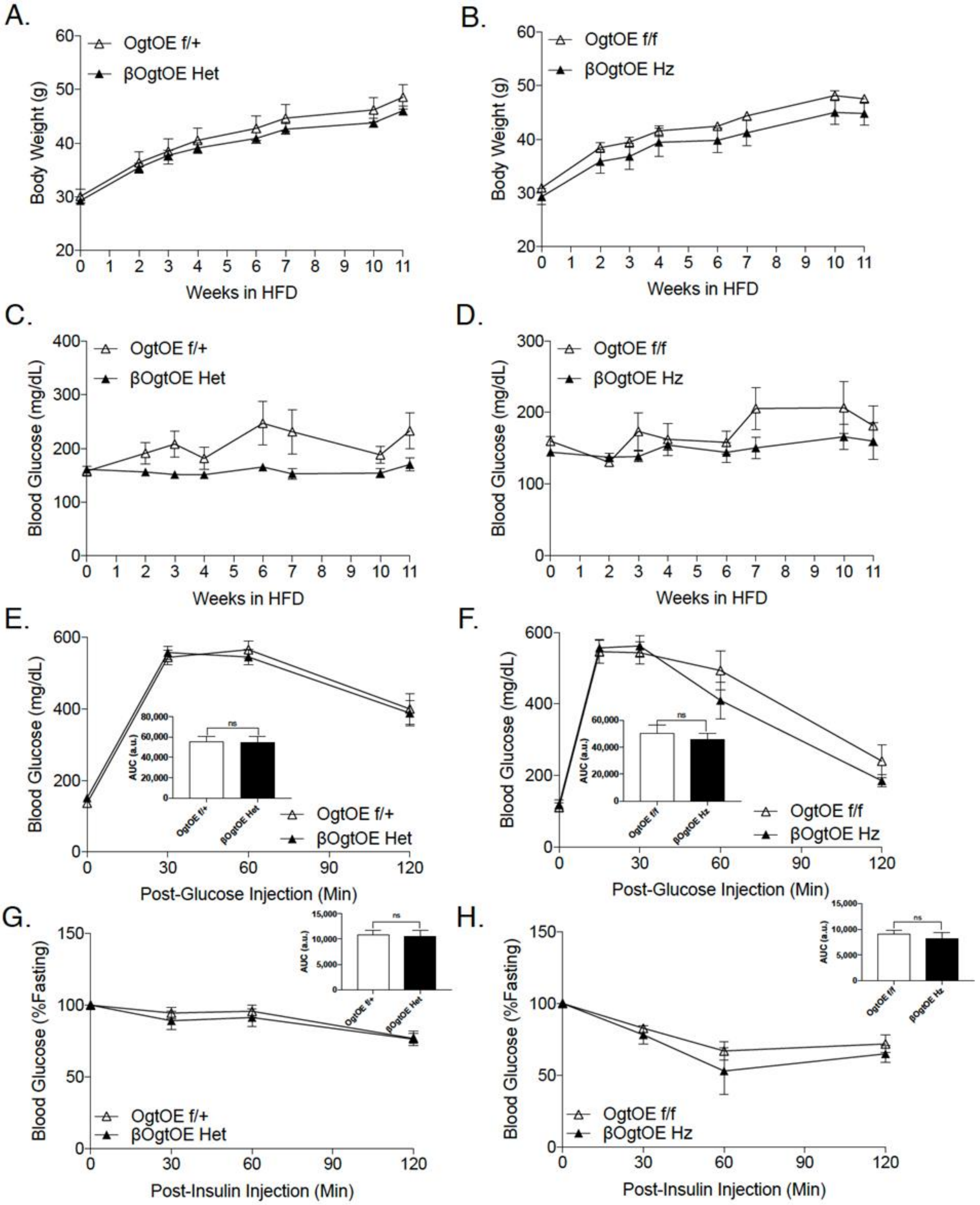

Supplemental Figure S4. Body weight over the course of 11 wks of high fat diet (HFD) in male βOgtOE Het (**A**) and βOgtOE Hz (**B**) with respective littermate controls. Random blood glucose over the course of 11 wks of HFD in βOgtOE Het (**C**) and βOgtOE Hz (**D**) with respective littermate (n = 10–16 βOgOE Het, n = 6 βOgtOE Hz). Intraperitoneal glucose Table 2. g/kg) from βOgtOE Het (**E**) and βOgtOE Hz (**F**), with littermate controls at 16-wks HFD (n = 10–16 βOgtOE Het, n = 6 βOgtOE Hz). Intraperitoneal insulin tolerance test (insulin 0.75 U/kg) from βOgtOE Het (**G**) and βOgtOE Hz (**H**), with littermate controls at 17-wks high fat diet (HFD) (n = 10–16 βOgtOE Het, n = 4–6 βOgtOE Hz). Statistical analyses were conducted using 2-way ANOVA with multiple comparisons, with significance $p < 0.05$, ns=non-significant.

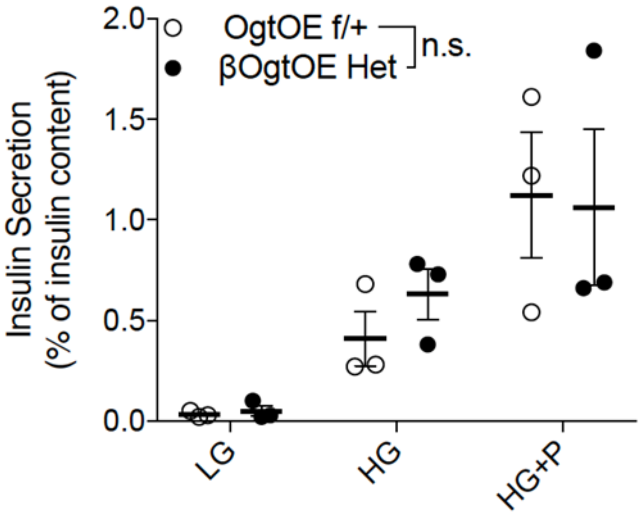

Supplemental Figure S5. In vitro insulin secretion in response to low glucose (LG, 3 mM) to high glucose (HG) (16.7 mM) with palmitate (100 uM; pre-complexed 6:1 to BSA, HG+*p*) in βOgtOE Het and control islets.

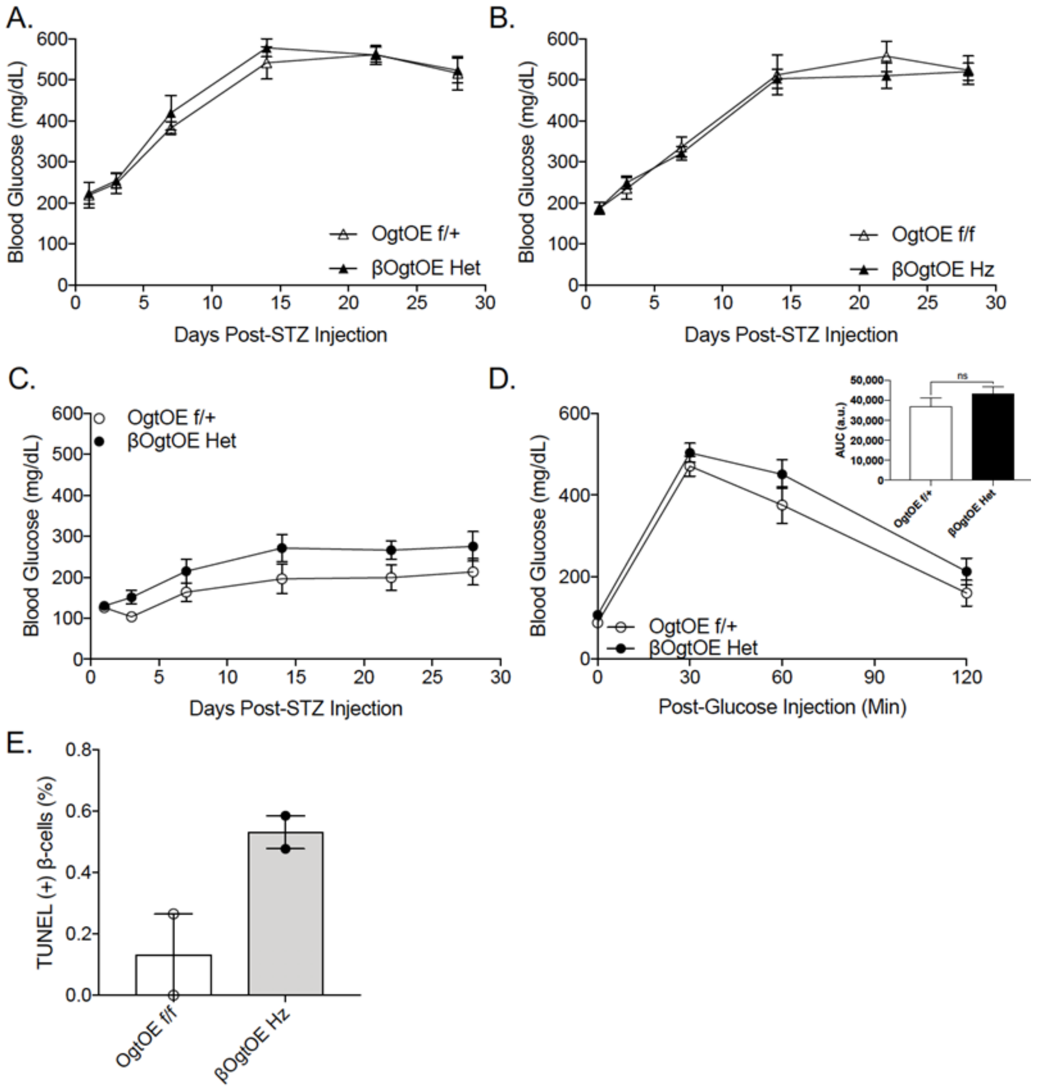

.

Supplemental Figure S6. Blood glucose over 4-wks post-STZ injection in male βOgtOE Het (**A**) and βOgtOE Hz (**B**) with littermate controls (*n* = 4–6). Blood glucose over 4-wks post-STZ injection (**C**) and intraperitoneal glucose tolerance test at 4-wks post-STZ in female βOgtOE Het (**D**) (*n* = 5). Apoptosis of β-cells were measured through TUNEL immunofluorescence staining in control and βOgtOE Hz pancreas (TUNEL (+) β-cells% to total β-cells counted) (**E**) (*n* = 2) , ns=non-significant.

A.

	βOgtOE Hz FC Value
GIP	30.960
PCDHB2	30.327
PDE6C	30.326
PCDH10	28.754
COL19A1	24.800
SLC45A1	24.165
CBLN1	22.669
CCDC125	22.134
Gm5088	19.167
MST1R	17.344
LYZL4	-17.365
CPXM1	-19.866
CENPI	-19.886
ART3	-19.895
LHX3	-20.163
GUCA2A	-25.309
IRAG1	-25.881
HYMAI	-27.149
RGSL1	-36.360
ACTN2	-38.764

B.

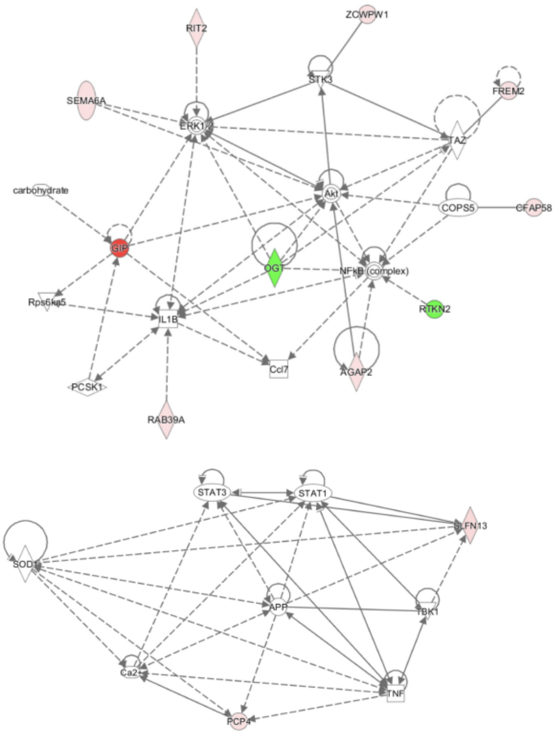

Supplemental Figure S7. (**A**) Top 10 upregulated and downregulated genes from βOgtOE Hz RNAseq. (**B**) Network analysis of the overlapping genes of interest from βOgtKO dataset, involving 10 (network 1) or 2 (network 2) sets of genes; red = upregulated, green = downregulated.

Table S1: Primer sequences list

Gene.	Primer 1	Primer 2
Actin	GCC CTG AGG CTC TTT TCC AG	TGC CAC AGG ATT CCA TAC CC
Rplp0	AGA TGC AGC AGA TCC GCA T	GTT CTT GCC CAT CAG CAC C
rOGT	TCG ATA GTC TAC CCG ATG TGA AGA	AGC TGT GTT AGT GGT GTC TGC ATT
mOGT	ACT GTG TTC GCA GTG ACC TG	TCA AAT AAC ATG CCT TGG CT
